# Gerontology Education in Social Work: The Role of Political Context and Alzheimer’s and Related Dementia Prevalence

**DOI:** 10.1093/geroni/igaf122.2945

**Published:** 2025-12-31

**Authors:** Grace Yi, Madelyn Martinez

**Affiliations:** California State University, Fullerton, Fullerton, California, United States; California State University, Fullerton, Fullerton, California, United States

## Abstract

As the aging population grows, there is an increasing need for professionals trained to address older adults’ diverse health and social needs. Social work, a discipline focused on supporting vulnerable populations, has played a key role in aging services. However, a shortage of professionals, including social workers, remains a concern. Limited research exists on the availability and content of gerontology courses in social work education programs. This current study aimed to examine factors influencing the inclusion of gerontology courses in social work programs and explore key themes in course content. A mixed-methods approach was applied. Using the list of CSWE-accredited MSW programs as a sampling framework, the top 150 universities ranked in U.S. News & World Report’s Best Schools for Social Work (44.1% of the total) were selected. The dataset included state-level factors (percentage of adults aged 65+, the prevalence of Alzheimer’s and related dementias (ADRD), political affiliation, and geographic region) and institutional characteristics. Aging course descriptions on institutions’ websites were analyzed qualitatively. Data analysis was conducted using Stata 18 and NVivo 14. While no direct association was found between state-level factors and course availability, a significant interaction effect was observed (B =-1.25, p =.03). Universities in Democratic-led states were more likely to offer courses as ADRD prevalence increased, whereas those in Republican-led states were less likely. Qualitative analysis identified key themes: family, care, clinical practice, and health. Findings highlight the role of the political climate in shaping gerontology education and the need to expand coursework to include macro-level practice.

